# Cutaneous Rhodococcus equi Infection in an Immunocompetent Host: A Case Report

**DOI:** 10.7759/cureus.101565

**Published:** 2026-01-14

**Authors:** Samantha Franco Gonzalez, Lucia Achell Nava, Sonia Toussaint Caire

**Affiliations:** 1 Dermatology, Centro Médico Nacional 20 de Noviembre, Instituto de Seguridad y Servicios Sociales de los Trabajadores del Estado, Mexico City, MEX; 2 Dermatopathology, Hospital General Dr Manuel Gea González, Mexico City, MEX

**Keywords:** actinobacteria, cutaneous bacterial infection, immunocompetent host infection, rare panniculitis, rhodococcus equi

## Abstract

*Rhodococcus equi* is a zoonotic Gram-positive bacterium that can affect immunocompetent hosts and only rarely presents with cutaneous lesions resembling other granulomatous dermatoses, such as cutaneous mycobacterial infections, deep fungal infections, sarcoidosis, and foreign body granulomas.

This report describes a 53-year-old immunocompetent woman who developed recurrent ulcerated nodules on the left lower extremity following recreational travel to southern Mexico. Initial diagnostic studies were nondiagnostic, and molecular analysis using polymerase chain reaction targeting the 16S rRNA gene confirmed infection with *Rhodococcus* spp. The patient received targeted antimicrobial therapy with subsequent clinical improvement.

This case supports considering *Rhodococcus equi* as a potential etiologic agent in persistent or relapsing granulomatous skin lesions, regardless of immune status, and demonstrates the utility of molecular diagnostics when conventional histopathologic assessment and culture-based methods fail to establish a definitive diagnosis.

## Introduction

*Rhodococcus equi *is a Gram-positive aerobic coccobacillus and a facultative intracellular pathogen, traditionally recognized as a zoonotic microorganism associated with pyogranulomatous pneumonia in foals, but with growing clinical relevance in human infections [1-9]. Initially regarded as an opportunistic pathogen predominantly affecting patients with advanced HIV infection or profound immunosuppression [2-7], an increasing number of cases in immunocompetent individuals have been reported in recent years, broadening the epidemiological and clinical spectrum of the disease [3,4,8]. This shift has led to the recognition of *R. equi* as an emerging pathogen capable of causing localized or systemic infections, including pulmonary disease, cutaneous infections, and bacteremia [2,4,6].

Clinical manifestations of *R. equi *infection are heterogeneous and largely depend on the host’s immune status. Immunocompromised patients most commonly develop cavitary pulmonary disease or disseminated infection, whereas immunocompetent individuals tend to present with focal forms, such as skin and soft tissue infections or chronic wound involvement [5,7,8]. Microbiological identification may be challenging due to variable morphology and slow growth in culture; however, molecular techniques such as 16S rRNA gene sequencing have significantly improved diagnostic accuracy, particularly in atypical or extrapulmonary cases [10].

Despite its low incidence, *R. equi* infection remains a diagnostic and therapeutic challenge because of its ability to survive within macrophages and its variable antimicrobial susceptibility profile [1,5]. The increasing number of reported cases in patients without traditional risk factors underscores the need to recognize its diverse clinical presentations and highlights the importance of obtaining a detailed clinical and exposure history to facilitate diagnosis, given the rarity of this pathogen. Consequently, *R. equi* should be included in the differential diagnosis of indolent or granulomatous infections [3,4,6].

## Case presentation

**Figure 1:**
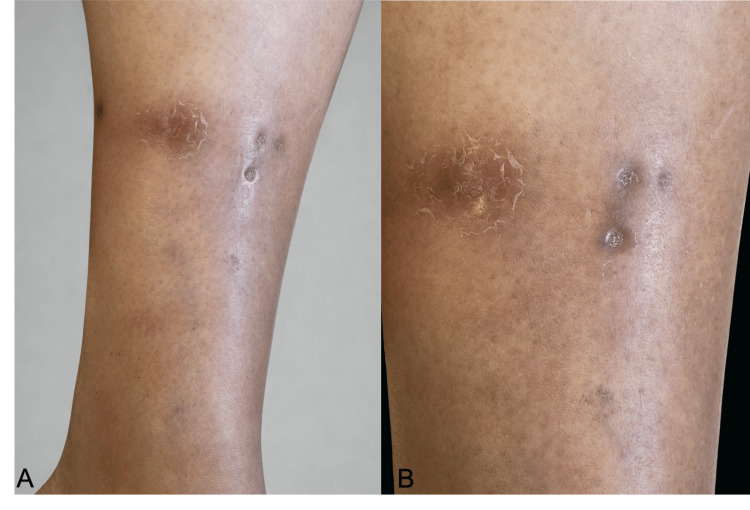
A and B. Violaceous subcutaneous nodular lesions alternating with punctate, round atrophic residual lesions following spontaneous ulceration.

A 53-year-old woman with no relevant chronic medical conditions presented with a history of recurrent cutaneous lesions. The initial episode developed shortly after a recreational trip to the state of Chiapas, Mexico. The primary lesion began as a papule on the left lower extremity that rapidly evolved over two weeks into a larger, erythematous, and painful nodule. On physical examination, inspection revealed an erythematous nodular lesion on the left lower extremity with central ulceration and purulent drainage. Palpation demonstrated a tender, indurated nodule without fluctuance. The lesion had evolved over approximately two weeks from an initial papule into a painful nodule, ultimately resolving spontaneously and leaving a small punctate atrophic scar (Figure 1).

Two months later, the patient reported the appearance of a second lesion adjacent to the original site, exhibiting identical clinical behavior, including rapid growth, burning pain at onset, suppuration, and spontaneous closure without residual scarring. Given the recurrent nature of the lesions, a skin biopsy and cultures were performed.

As conventional microbiological studies were inconclusive, histopathologic examination demonstrated mild epidermal acanthosis with focal spongiosis and basal hyperpigmentation, accompanied by a mild perivascular lymphocytic infiltrate in the dermis. The subcutis showed a dense mixed inflammatory infiltrate with multinucleated giant cells surrounding neutrophilic microabscesses, consistent with septal and focal lobular panniculitis.

Key differential diagnoses, including cutaneous mycobacterial and deep fungal infections, were subsequently ruled out. During this period, only supportive topical management with emollients and a re-epithelializing cream was provided while awaiting molecular diagnostic results.

**Figure 2:**
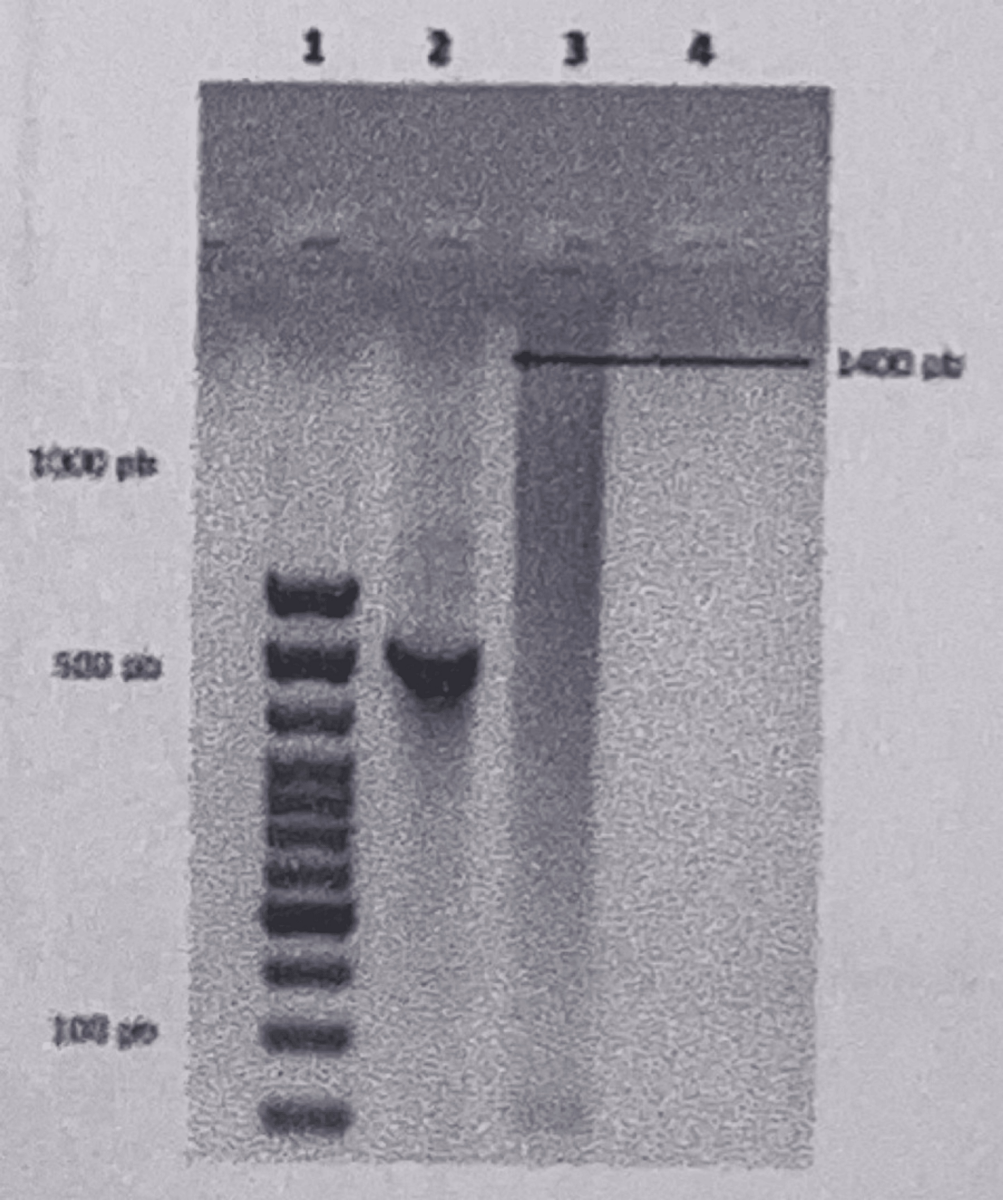
PCR cassette showing a positive amplification of the 16S rRNA gene specific to the Actinobacteriaceae family, confirming Rhodococcus infection.

Molecular testing was then pursued, and polymerase chain reaction (PCR) amplification targeting the 16S rRNA gene confirmed infection with *Rhodococcus *spp. (Figure 2). Following diagnostic confirmation, targeted antimicrobial therapy with azithromycin and rifampicin was initiated for eight weeks, as recommended by the infectious diseases service, resulting in complete clinical resolution.

## Discussion

Human infection caused by *Rhodococcus *spp., members of the family Actinobacteriaceae, is uncommon. A major diagnostic challenge lies in its ability to mimic other chronic granulomatous infections, such as mycobacterial disease, actinomycosis, or cutaneous leishmaniasis. In the present case, the patient exhibited recurrent cutaneous lesions with histopathological findings of granulomatous and suppurative panniculitis, a pattern previously described in infections caused by actinobacteria, including *R. equi* [1,2].

Molecular identification using PCR targeting the 16S rRNA gene, revealing an amplicon of approximately 1,400 base pairs consistent with Actinobacteriaceae, was pivotal in establishing the diagnosis [10]. This finding is particularly relevant, as *R. equi* may be misidentified as commensal flora or confused with acid-fast organisms, leading to diagnostic delays, as widely reported in the literature [1,3,4].

Although most human *R. equi* infections occur in immunocompromised hosts - especially patients with advanced HIV infection, hematologic malignancies, or solid organ transplants [1,5,6] - cases in immunocompetent individuals have been documented, typically following environmental exposure or direct inoculation through contaminated wounds [4,7]. In this case, recent travel to Chiapas, a region where exposure to moist soil, dust, and potential animal reservoirs is common, represents a relevant epidemiological factor. Cutaneous inoculation has been described in cases of cellulitis, granulomatous mastitis, and panniculitis caused by *Rhodococcus* in immunocompetent hosts [4,7,8].

The presence of granulomatous and suppurative panniculitis aligns with the known pathophysiology of *Rhodococcus*, which is characterized by intracellular survival within macrophages and a chronic inflammatory response with granuloma formation and suppurative foci [2,3]. However, these histological features are not specific and may also be observed in infections caused by mycobacteria or *Nocardia*, reinforcing the importance of molecular techniques when cultures are negative or morphology is nonspecific. In this case, 16S rRNA PCR was essential, consistent with studies highlighting its utility in detecting slow-growing or difficult-to-identify actinobacteria [9,10].

Diagnostic delays are frequently reported due to the rarity of the infection and its clinical resemblance to other chronic granulomatous dermatoses [1,4]. Nevertheless, unlike disseminated disease in immunocompromised patients - where mortality rates of up to 50% have been reported [5,6] - localized cutaneous infections in immunocompetent individuals generally have a more favorable prognosis when diagnosed and treated promptly [4,7].

Treatment poses additional challenges due to limited data and variable antimicrobial susceptibility, making in vitro susceptibility testing essential. Resistance mechanisms, including β-lactamase production, have been described, rendering β-lactam monotherapy controversial unless combined with β-lactamase inhibitors. *Rhodococcus* species are typically susceptible in vitro to macrolides, rifampin, aminoglycosides, imipenem, and vancomycin. Combination therapy with two or three antibiotics is recommended to prevent resistance, particularly agents with good intracellular penetration such as rifampin and azithromycin, given the organism’s intracellular localization. Treatment duration depends on disease severity and immune status: mild localized infections in immunocompetent patients may respond to two to eight weeks of oral therapy, whereas immunosuppressed or transplant recipients often require prolonged and aggressive regimens [1].

## Conclusions

*Rhodococcus* spp. infection remains a rare entity in humans and a diagnostic challenge due to its ability to mimic other chronic granulomatous dermatoses. This case demonstrates that, even in immunocompetent individuals, recent environmental exposure can facilitate cutaneous inoculation and result in localized disease with histopathological findings of granulomatous and suppurative panniculitis. Molecular techniques, particularly 16S rRNA gene amplification, were crucial in guiding diagnosis in the setting of nonspecific clinical and histological features, reaffirming their value in infections caused by difficult-to-identify actinobacteria. The integration of clinical, epidemiological, histopathological, and molecular data allowed recognition of an uncommon presentation of *Rhodococcus* infection, expanding its known clinical spectrum and emphasizing the need to include it in the differential diagnosis of chronic granulomatous skin lesions in immunocompetent hosts.
